# Research Progress in the Extraction, Structural Characteristics, Bioactivity, and Commercial Applications of Oat β-Glucan: A Review

**DOI:** 10.3390/foods13244160

**Published:** 2024-12-22

**Authors:** Xiaolu Li, Yicheng Wu, Ruilin Duan, Haoran Yu, Siyao Liu, Yulong Bao

**Affiliations:** 1 School of Food and Biological Engineering, Jiangsu University, Zhenjiang 212013, China; 2 School of Pharmacy, Jiangsu University, Zhenjiang 212013, China

**Keywords:** oat, β-glucan, bioactivities, processing, application

## Abstract

Oats (*Avena sativa* L.) are an important cereal crop with diverse applications in both food and forage. Oat β-glucan has gained attention for its beneficial biological activities, such as reducing cardiovascular risk, preventing diabetes, and enhancing intestinal health. Despite its potential, more comprehensive research is required to explore its preparation, modification, bioactivities, and applications. This review highlights recent advancements in the determination and preparation of oat β-glucan, explores its biological activities and mechanisms, and examines the impact of food processing techniques on its properties. This review is intended to provide a theoretical foundation and reference for the development and application of oat β-glucan in the functional food industry.

## 1. Background

**Figure 1:**
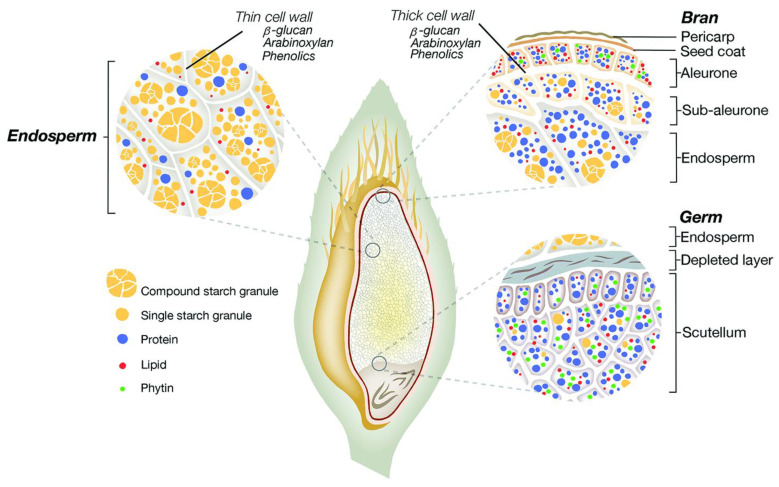
Structural representation of the oat grain presenting different oat tissues and the nutrient distribution [3].

Oats (*Avena sativa* L.) are an ancient cereal crop recognized for their dual role as both a staple grain and a significant source of forage [1]. The cultivation of oats is concentrated in temperate regions of the Northern Hemisphere, particularly in countries such as Russia, Canada, the United States, Australia, Germany, Finland, and China [2]. Structurally, oats comprise three main components: bran, endosperm, and germ, as depicted in Figure 1. The pericarp constitutes the outermost layer of the oat bran, while the innermost layer is the endosperm. Intermediary layers include the seed coat, aleurone layer, and sub-aleurone layer [3]. Notably, the endosperm accounts for approximately 84% of the oat grain and is rich in essential nutrients, including starch, protein, and lipids [4]. Additionally, the cell walls of the aleurone and endosperm layers are abundant in oat β-glucan [5]. The β-glucan content in oats typically ranges from 4% to 10%, which exceeds the levels found in barley and wheat. This variation in β-glucan content is influenced by several factors, including the specific oat and barley varieties, environmental growing conditions, and the timing of harvest [4].

Oat β-glucan is a linear polysaccharide primarily composed of glucose units linked by β-(1→4) bonds, with β-(1→3) linkages occurring every 2 to 3 units. It contains approximately 70% β-(1→4) linkages and 30% β-(1→3) linkages, exhibiting a molecular ratio of 1.5 to 2.1 and a molecular weight ranging from 6.5 × 10^4^ to 3.1 × 10^6^ Da [6]. Due to its advantageous properties, such as excellent water solubility, high viscosity, and gel-forming ability, oat β-glucan is widely utilized in both the food and pharmaceutical industries [7]. Its stability under various conditions, including heat, acid, and alkali, renders it suitable as an emulsifier, thickener, stabilizer, and natural preservative in food formulations [8].

Oat β-glucan has been incorporated into a variety of food products, including wheat bread, porridge, wheat noodles, low-fat beef patties, and dairy products [9,10,11]. Research into oat β-glucan has attracted considerable attention due to its multiple beneficial biological functions, establishing it as a prominent area of study worldwide. Notably, in the 1990s, the United States Food and Drug Administration (FDA) reported that a daily intake of over 3 g of β-glucan or the consumption of low-fat oats can lower blood lipid levels and reduce the risk of cardiovascular diseases [12]. This endorsement has significantly accelerated the scientific investigation and commercial applications of oat β-glucan. Numerous studies have confirmed its hypoglycemic effects, potential to improve gut microbiota, antioxidant properties, and immune-enhancing effects [13]. This review aims to provide a comprehensive overview of recent advancements in the detection, separation, purification, structural characterization, and biological activity of oat polysaccharides. The current review also delves into the underlying relationship between structural characteristics and bioactivities, offering insight into the future development trends of oat polysaccharides, as well as ultimately advancing their applications in the medicinal and functional food industries.

## 2. Determination of Oat β-Glucan

β-Glucan is widely utilized in the food industry and possesses significant physiological activity. Therefore, determining the β-glucan content is crucial at various stages, including breeding, processing, and product development. Common detection methods for β-glucan include enzymatic assays, high-performance liquid chromatography (HPLC), and Congo red assays [14]. These methods can be categorized into two primary principles: one involves hydrolyzing high-molecular-weight β-glucan molecules into individual glucose units, with the β-glucan content quantified based on glucose concentration; the other employs dyes (such as Congo red) that specifically bind to β-glucan, quantifying it through changes in spectral absorbance.

### 2.1. Enzymatic Assay

**Figure 2:**
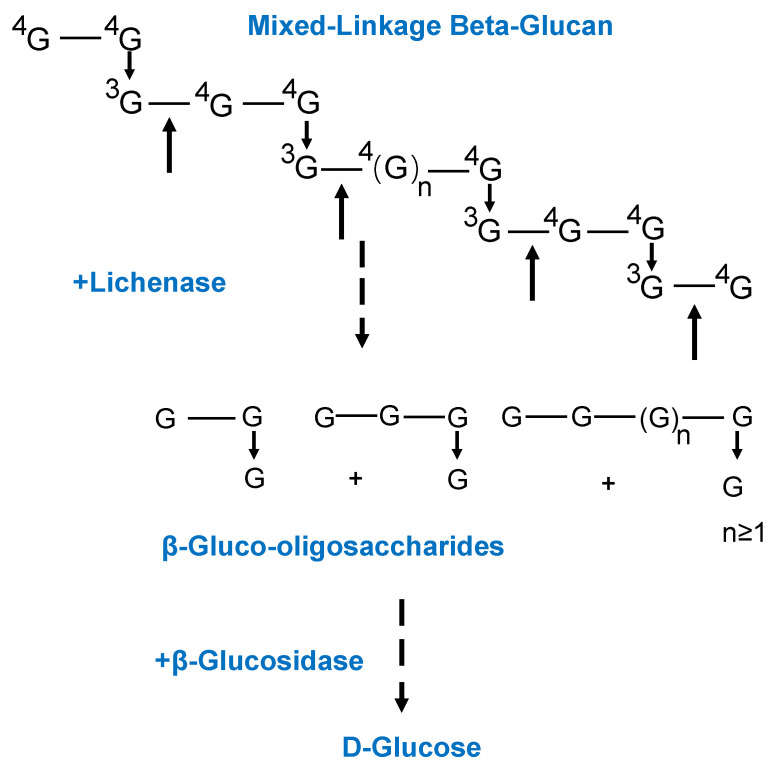
Principle of the enzymatic analysis of β-glucan [18].

The most widely employed β-glucan detection method is McCleary’s enzymatic assay, also referred to as the β-glucan reagent kit method [15]. Introduced by McCleary in 1985, this method continues to serve as the standard for detecting β-glucan in barley, oats, malt, and beer. It has received certification from authoritative organizations, including the Association of Official Analytical Chemists (AOAC) and the American Association of Cereal Chemists (AAC). The principle of this method for detecting β-glucan in grains is illustrated in Figure 2. Initially, the ground grain sample undergoes pre-extraction to isolate β-glucan, which is then hydrolyzed into oligosaccharides by lichenase. Subsequently, β-glucanase breaks these oligosaccharides down into individual glucose molecules. A glucose peroxidase buffer is added, and the glucose concentration is quantitatively assessed at a wavelength of 510 nm, facilitating the calculation of β-glucan content [16]. This method is known for its high accuracy, established operational protocols, and stable results, making it the most widely utilized technique for β-glucan detection. However, the high cost of highly purified and specific enzymes can be a limiting factor for large-scale testing. While McCleary’s method is a widely accepted standard, US 11913060 B1 discloses alternative methods for quantifying mixed-linkage β-glucan that focus on improved alkaline pretreatment of the sample prior to enzymatic hydrolysis and glucose measurement [17]. These methods aim to enhance the accuracy, precision, and sensitivity of β-glucan assays through optimized parameters for each step, including the use of a specific conversion factor for glucose to β-glucan calculation.

### 2.2. Chromatographic Analysis

β-Glucan detection can also be accomplished through chromatographic methods. This approach relies on the hydrolysis of β-glucan using hydrochloric acid (HCl), trifluoroacetic acid (TFA), or sulfuric acid (H_2_SO_4_) at elevated temperatures. The monosaccharides and oligosaccharides generated from this acid hydrolysis can subsequently be analyzed quantitatively using gas chromatography (GC) or high-performance liquid chromatography (HPLC) [19]. Like the enzymatic method, this chromatographic approach necessitates the extraction of β-glucan prior to hydrolysis with strong acids at high temperatures. The resulting monosaccharides are then quantitatively analyzed using HPLC. Although this method is highly accurate, it involves a complex sample preparation process. Moreover, the use of concentrated acids at elevated temperatures introduces significant safety risks [20].

### 2.3. Fluorescence Method

The fluorescence method operates on the principle of specific binding between the fluorescent whitening agent calcofluor and β-glucan, which allows for quantification based on variations in fluorescence intensity [21]. Flow injection analysis (FIA) was developed in the 1980s to meet the demand for a simple, accurate, and rapid detection method for β-glucan in beer [22]. Researchers designed an automated FIA system that exploits the specific binding of calcofluor to water-soluble high-molecular-weight β-glucan for measuring its concentration [23]. This method utilizes a standard β-glucan of known purity for calibration; the fluorescence intensity measured is then compared to a calibration curve to quantify the β-glucan content. However, the precise interaction mechanism between calcofluor and β-glucan remains poorly understood. Additionally, variations in the molecular weight of β-glucan can affect fluorescence intensity, while factors such as ionic strength and the presence of other substances in the measurement system may also influence the outcomes [23]. Therefore, a systematic evaluation is necessary to accurately determine β-glucan content in oats using this method.

### 2.4. Staining Method

Congo red dye has been shown to bind to β-glucan, and several studies have demonstrated its effectiveness in detecting β-glucan content in grains [24,25]. When β-glucan is present, the absorbance of Congo red at 550 nm—measured using a UV–visible spectrophotometer—correlates with the concentration of β-glucan. By establishing a standard curve with a known concentration of β-glucan, it is possible to quantify the β-glucan content in various samples. This method offers several advantages, including rapidity and simplicity, and it allows for the simultaneous analysis of multiple samples using a 96-well plate format [26]. Additionally, the relatively low cost of Congo red dye enhances its economic feasibility for large-scale applications. However, the complex composition of grain samples can affect the interaction between Congo red and β-glucan [24], as other components may interfere with the measurement [27]. In particular, certain water-soluble polysaccharides present in oats can diminish the overall precision and reliability of the method. While Congo red is a common dye used for β-glucan detection, EP 2810051 A1 describes a method using calcofluor dyes, such as Calcofluor White or Calcofluor White M2R, for selectively determining β-glucan concentrations in samples, particularly liquid samples of cereal origin [28]. This patent highlights the use of photometric measurement of the dye–β-glucan complex, similar to the Congo red method, but with an alternative dye.

### 2.5. Enzyme-Linked Immunosorbent Assay

The enzyme-linked immunosorbent assay (ELISA) is an effective technique for detecting β-glucan content [29]. This method employs specific antibodies that bind to β-glucan, allowing for the quantitative measurement of soluble β-glucan in oats at trace levels through a colorimetric reaction [30]. Rampitsch et al. [31] developed 31 monoclonal antibodies and conjugated β-glucan with keyhole limpet hemocyanin to assess the cross-reactivity of these antibodies with both β-glucan and other polysaccharides. The results indicated strong reactivity of the selected antibodies with β-glucan from oats. Additionally, the study optimized the extraction protocols for β-glucan from oat samples, as well as the ELISA detection procedures, improving the repeatability, accuracy, and throughput of the method. A high correlation (r > 0.9) was observed when comparing ELISA results with those obtained from enzymatic methods. The ELISA technique is theoretically applicable to any product containing β-glucan, including oat-based foods, oat grains, and bran. However, the high sensitivity of the assay, driven by the strong antibody interactions, may impact measurement accuracy [32]. Thus, further evaluation of the accuracy and applicability of this method is necessary.

## 3. Separation and Purification of Oat β-Glucan

Oat β-glucan extraction methods can be broadly categorized into dry and wet extraction techniques [33]. While dry extraction methods, such as grinding and sieving, offer the advantage of preserving the native structure and function of β-glucan, they often exhibit limitations in terms of yield and require multiple processing steps [34]. Air classification, a commonly employed dry extraction method, has shown effectiveness in enriching β-glucan content [35]. The optimized air classification method involved a multi-step process, beginning with dehulling and followed by soft pearling. These pretreatments effectively removed the hulls and outer layers of the kernel, which contain insoluble dietary fiber, thereby optimizing the yield and β-glucan enrichment during subsequent air classification. Despite these optimizations, the overall yield of this process remained relatively low, typically ranging from 3.7 to 8.3 g/100 g of barley flour, with an overall product yield generally below 20% [36].

In recent years, electrostatic separation has emerged as a promising alternative to traditional dry extraction methods. In contrast to air classification, which primarily relies on particle size and density differences, electrostatic fractionation leverages the distinct dielectric properties of particles [37]. This enables the effective separation of particles that may be similar in size and density but exhibit differing surface charges, a distinction that is not readily achieved through traditional methods like air classification or sieving. Sibakov et al. [37] demonstrated that combining ultrafine grinding with electrostatic separation can significantly enhance β-glucan yields, achieving a remarkable 56.2% β-glucan content in oat extracts. This finding underscores the potential of electrostatic fractionation as a more efficient and effective method for enriching β-glucan compared to conventional dry extraction techniques [38]. Moreover, electrostatic separation offers the potential for energy efficiency, further enhancing its appeal as a sustainable approach.

Wet extraction, which encompasses solvent extraction techniques, includes water extraction, alkaline extraction, enzymatic extraction, and subcritical extraction [36]. These methods can be used individually or in combination, and can also be augmented with additional techniques such as ultrasound, microwave, or pulsed electric fields. In contrast to dry extraction, wet extraction is influenced by various factors, including the type and concentration of the solvent, temperature, duration, pH, stirring conditions, particle size, and the composition of the raw materials [36].

### 3.1. Pretreatment

Enhancing the extraction efficiency of β-glucan typically requires pretreatment of raw materials. Dry grinding and sieving serve as effective preparatory steps for subsequent wet extraction. Additionally, methods such as roasting, steaming, baking, extrusion puffing, and homogenization can significantly affect the extraction rate of oat β-glucan [39]. Research demonstrates that extrusion treatment yields the highest extraction rate, followed by steaming and baking [40]. Before proceeding with wet extraction, it is essential to perform defatting and inactivate enzyme activity to further enhance extraction efficiency and purity [41,42]. Common defatting solvents include petroleum ether, ether, ethanol, and isopropanol. Notably, during the defatting process using ethanol, maintaining a temperature of 80 °C effectively inactivates endogenous β-glucanase while also removing small molecules, sugars, proteins, and lipid-soluble substances [39].

### 3.2. Water Extraction

Oat β-glucan is insoluble in organic solvents such as alcohols, ethers, and ketones, but readily soluble in water, which makes hot water extraction an effective approach. Studies indicate that extracting β-glucan from oat bran with hot water yields the highest β-glucan content (5.3%), in comparison to lower yields achieved through enzymatic, acidic, or alkaline methods [43]. Wang et al. [44] employed a water extraction technique on concentrated oat bran, where the conventional process produced a product containing 66% β-glucan. Acidifying the extract to pH 3 before ethanol precipitation increased the β-glucan content to 69%, and subsequent removal of phytic acid raised the concentration to 72.7%. In comparison, researchers used a combination of hot water immersion and freeze–thaw cycling to extract oat β-glucan without inactivating endogenous enzymes, conducting the extraction at 55 °C for 2 h. The resulting extract was concentrated to achieve a β-glucan mass fraction of 1%. Following three freeze–thaw cycles, the yield was recorded at 1.5%, with a purity of 92% [45]. A recent patent describes a water extraction method combined with a specific filtration process to obtain an oat extract enriched in both avenanthramides and β-glucan, highlighting the potential for co-extraction of these valuable oat components [46]. While the water extraction method utilizes milder conditions, the prolonged extraction time increases operational costs. Additionally, the substantial volume of solvent required, along with the need for solvent recovery, contributes to higher energy consumption.

### 3.3. Alkaline Extraction

The alkaline extraction method is effective for certain acidic or high-molecular-weight β-glucans that are insoluble in hot water but soluble in dilute alkaline solutions. Chaiyasut et al. [47] reported extracting total glucans from oat samples using a 1.0 mol/L NaOH solution, yielding an extract with a total glucan content of 89%, of which β-glucan accounted for 84%. Researchers compared extraction methods, employing carbonate, 0.05 mol/L NaOH, and boiling water with heat-stable α-amylase, and found that the alkaline methods achieved β-glucan extraction rates between 86% and 98%, while hot water extraction resulted in lower yields of 36% and 28%, respectively [6]. Additionally, a study optimized extraction conditions using a dilute alkaline solution at pH 10.9 for 1.9 h, achieving a β-glucan yield of 4.36% [45]. Despite its relatively high extraction yield, alkaline extraction has received limited attention in recent years. This method may lead to partial depolymerization of β-glucan molecules. Moreover, alkaline extraction often increases protein and starch contamination, which complicates subsequent purification and decolorization processes [48].

### 3.4. Enzymatic Extraction

**Figure 3:**
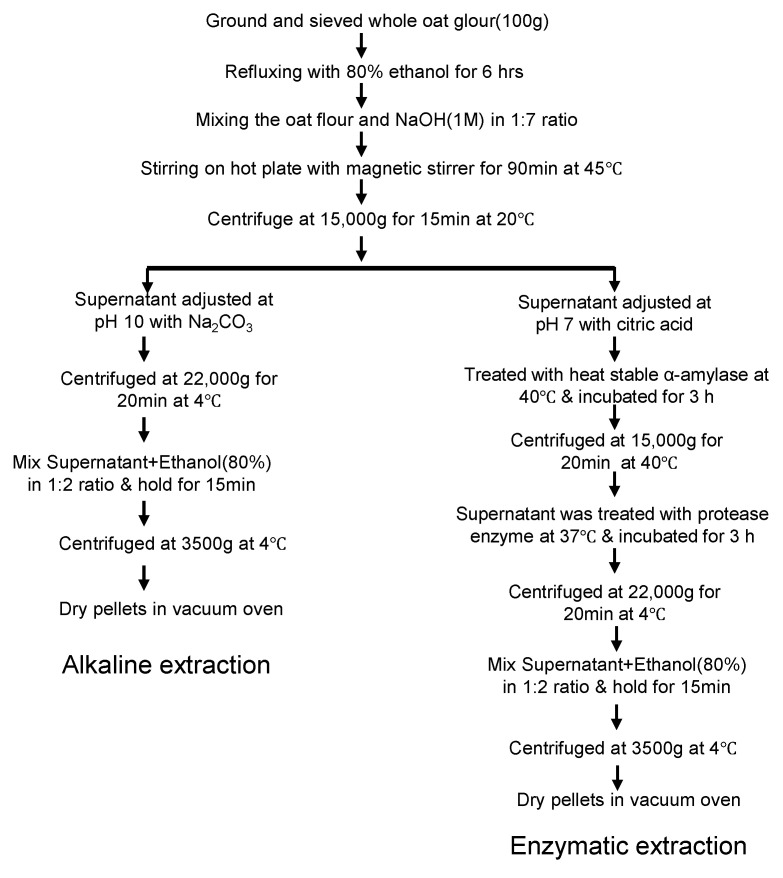
Extraction and purification scheme of β-d-glucan [41].

The enzymatic extraction method takes advantage of enzyme specificity to selectively degrade and remove impurities from the extract, resulting in a high-purity product (Figure 3). Studies showed that oat gum yields from enzymatic, alkaline, and acidic extraction range from 3.74% to 5.14%, with enzymatic extraction providing the highest yield at 5.14%. For β-glucan, the extraction rate ranges from 82.1% to 86.8%, with the highest rate achieved by the enzymatic method (86.8%) and the lowest by the alkaline method, likely due to enzymes being more effective at removing starch and protein [41].

Further research highlights that enzymatic extraction produces β-glucan with a higher molecular weight, greater yield, stable colloidal properties, and lower protein content, achieving a yield of 13.9%, compared to 6.97% and 5% for acidic and alkaline extraction methods, respectively [49]. Mishra et al. [50] compared β-glucan extraction using alkaline, acidic, hot water, and enzymatic methods. They found that an enzymatic method using heat-stable α-amylase and protease provided the highest extraction yield (86.7%) and exhibited superior antioxidant and antibacterial activities. A patent application describes an enzyme preparation specifically designed for producing β-glucan-containing plant-based liquid compositions, further emphasizing the potential of enzymatic methods for efficient and targeted β-glucan extraction. This preparation utilizes a combination of α-amylase and β-glucanase to optimize the extraction process and potentially enhance the functional properties of the resulting β-glucan [51]. Compared to chemical reagent-based methods, enzymatic extraction is safer, more environmentally friendly, and results in a purer product. This method can also replace certain chemical processes, enhancing overall extraction efficiency.

### 3.5. Subcritical Water Extraction

Subcritical water extraction involves using water under subcritical conditions as the solvent [52]. With these conditions, water’s viscosity decreases while its diffusion coefficient increases, facilitating deeper penetration into the sample matrix and accelerating the extraction of β-glucan [53,54]. Yoo et al. [55] demonstrated that extracting β-glucan from oat flour under specific conditions—200 °C, pH 4.0, for 10 min, with a particle size range of 425–850 μm—resulted in a yield of 6.98% and an extraction efficiency of 88.08%. This efficiency was significantly higher than that obtained through hot water extraction, which reached only 36.62%. In pilot-scale trials, optimal extraction conditions were found at 210 °C for 10 min, yielding 3.01% β-glucan with an efficiency of 76.36%. Du et al. [56] employed accelerated solvent extraction to isolate β-glucan from bran, optimizing parameters at 70 °C for 9 min, with four extraction cycles at 10 MPa. This process achieved a yield of 16.39%. Compared to traditional solvent extraction techniques, subcritical extraction offers several advantages, including higher β-glucan yields, reduced environmental impact, shorter extraction times, and minimal degradation. These attributes make subcritical extraction highly suitable for industrial-scale applications [57].

### 3.6. Removal of Starch and Protein

High-temperature extraction methods—such as water extraction, alkaline extraction, and subcritical extraction—frequently cause starch gelatinization, leading to the co-extraction of starch with β-glucan, which diminishes its purity. To enhance the purity of β-glucan, industrial production typically employs a stepwise enzymatic hydrolysis process. Initially, α-amylase degrades starch into dextrins, which are subsequently converted into glucose by glucoamylase and then removed via dialysis. Papageorgiou et al. [58] demonstrated that treatment with heat-stable α-amylase at 90 °C for 3 h (pH 4.5) effectively eliminated starch, resulting in a nearly starch-free final product.

Protein is another major impurity in crude β-glucan extracts. Unlike starch removal, protein elimination offers a broader range of methods, including the Sevag method [59], enzymatic hydrolysis [60], isoelectric precipitation [61], and combinations of enzyme-based strategies. Harasym et al. [48] employed alkaline extraction followed by trypsin, heat-stable α-amylase, and isoelectric precipitation, achieving a β-glucan purity of 97% for both high- and low-molecular-weight fractions. Further purification using a combination of trypsin, heat-stable α-amylase, glucoamylase, and papain increased β-glucan contents to 97.5% and 99.3%, respectively. The combination of α-amylase for starch hydrolysis and trypsin-isoelectric precipitation for protein removal is widely used in initial purification processes, as it achieves high removal rates and excellent β-glucan retention compared to other methods.

### 3.7. Removal of Pigments and Small Molecule Impurities

Pigments in the extract can compromise product quality, making decolorization essential [62]. Activated carbon is frequently used for its efficacy in removing both pigments and proteins. Alternative materials include diatomaceous earth, cellulose, hydrogen peroxide, macroporous adsorption resin, resin–carbon combinations, and ion exchange columns (e.g., DEAE-cellulose) [63,64]. Among these, macroporous adsorption resin demonstrates superior β-glucan retention compared to activated carbon during decolorization [65]. Small molecule impurities and heteropolysaccharides present in the extract are typically removed using precipitation or membrane separation techniques. Common precipitants include ethanol, acetone, isopropanol, and ammonium sulfate. Ryu et al. [66] extracted β-glucan from oats with a Na₂CO₃ solution at 45 °C, followed by purification with 300 g/L ammonium sulfate and 50% (*v*/*v*) isopropanol, achieving a yield of 1.9% and a purity of 78.8%. In another study, β-glucan was extracted by water at sub-gelatinization temperatures, followed by enzymatic starch hydrolysis, protein removal at pH 4.0–4.5, and final precipitation with 80% (*v*/*v*) ethanol, resulting in a purity of 90.4% to 93.7% and a molecular weight of 0.44–1.10 × 10^5^ Da [58]. Ethanol precipitation stands out as the most effective purification method, efficiently concentrating β-glucan while also removing proteins, lipids, and pigments, making it a versatile choice for high-purity extraction.

## 4. The Bioactivities of Oat β-Glucan

**Figure 4:**
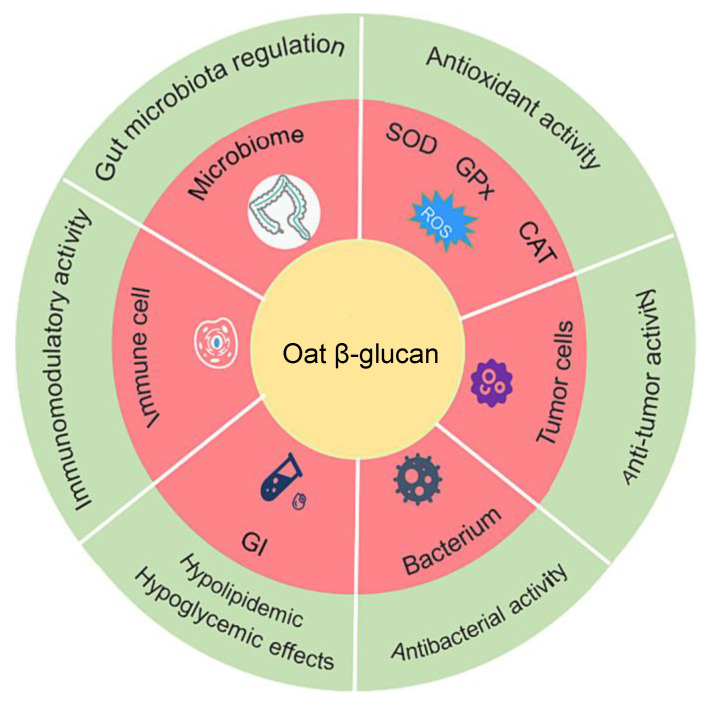
Summary of the mechanisms of biological activities of oat β-glucan [65].

Oat β-glucan has emerged as a promising functional ingredient for promoting health and preventing a variety of diseases. It plays a key role in supporting gut health by normalizing the gut microbiota, which can contribute to improved overall metabolic function [67]. Its ability to help prevent diabetes is linked to its effects on blood glucose management, as it lowers postprandial blood glucose levels and reduces insulin response, thereby enhancing glycemic control. Additionally, oat β-glucan has been found to lower cholesterol and blood pressure, both of which contribute to a reduced risk of cardiovascular diseases [1]. It also has appetite-regulating properties, which can support weight management and mitigate diabetes-related complications [68]. The beneficial effects of oat β-glucan extend to enhancing postprandial satiety, likely due to its resistance to digestion in the gastrointestinal tract, leading to prolonged fullness [69]. Furthermore, it has shown potential in reducing abdominal fat and addressing obesity, as demonstrated by significant decreases in body weight, body mass index, and body fat percentage [70]. Figure 4 offers a comprehensive summary of the bioactivities of oat β-glucan.

### 4.1. Hypoglycemic Effects

Diabetes is characterized by chronic hyperglycemia, leading to symptoms such as excessive thirst, increased hunger, frequent urination, and weight loss [71,72]. Oat β-glucan has been widely studied for its potential to manage diabetes, as it is known to lower cholesterol and triglycerides and stabilize blood glucose levels. Research suggests that β-glucan may exert its hypoglycemic effects by modifying the microstructure of food, limiting starch gelatinization, and reducing α-amylase hydrolysis [73]. Dong et al. [74] found that oat β-glucan regulates glucose metabolism and inhibits α-glucosidase activity, suggesting its potential as an α-glucosidase inhibitor. Shen et al. [75] demonstrated that oat β-glucan reduces fasting blood glucose and glycated serum protein in diabetic mice by enhancing GLP-1 secretion, promoting glycogen synthesis, and activating key enzymes in the tricarboxylic acid cycle.

Studies have shown that oat β-glucan enhances the activity of intestinal Na^+^-K^+^-ATPase and Ca^2+^-Mg^2+^-ATPase, improves metabolism, and reduces insulin resistance, which contributes to better blood glucose control [76]. Hooda et al. [77] reported that adding 6% concentrated oat β-glucan to the diet reduced peak blood glucose and insulin levels in pigs while increasing short-chain fatty acid (SCFA) production. Liu et al. [78] observed that oat β-glucan improves hepatic glucose metabolism and restores pancreatic β-cell integrity in diabetic mice. Abbasi et al. [79] found that oat β-glucan inhibits glucose uptake and the expression of glucose transporters SGLT1 and GLUT2 in IEC-6 cells. Wang et al. [80] showed that the combined use of oat β-glucan and L-arabinose improved glucose uptake in insulin-resistant cells by activating the PI3K/AKT signaling pathway. Overall, oat β-glucan exerts its hypoglycemic effects by reducing glucose absorption in the gastrointestinal tract, regulating hormone levels, promoting SCFA production, supporting pancreatic health, modulating glucose transport, activating key signaling pathways, and enhancing the growth of beneficial gut bacteria (Table 1).

### 4.2. Cholesterol-Lowering Effects

Both the United States Food and Drug Administration (FDA) and the European Food Safety Authority (EFSA) recommend a daily intake of 3 g of oat or barley β-glucan to reduce blood cholesterol levels and the risk of coronary heart disease [83,84]. Studies have demonstrated that consuming at least 3 g/day of oat β-glucan can reduce total plasma cholesterol and low-density lipoprotein (LDL) cholesterol by 5% to 10% in individuals with normal or elevated cholesterol levels. Higher intakes, exceeding 3 g/day, have been shown to decrease total and LDL cholesterol by 0.25 mmol/L and 0.30 mmol/L, respectively, without affecting high-density lipoprotein (HDL) cholesterol or triglyceride levels [84]. Ho et al. [12] also reported reductions in LDL and non-HDL cholesterol, indicating that β-glucan-rich foods may reduce cardiovascular disease risk.

Hasan et al. [85] found that the cholesterol-lowering effects of oat β-glucan are associated with its molecular properties, such as weight-average molecular weight, solubility, and viscosity. The mechanism behind β-glucan’s effect on cholesterol remains partially understood; current evidence suggests that it may reduce cholesterol by interfering with bile acid recirculation and influencing cholesterol metabolism [86,87]. Miyamoto et al. [88] proposed that oat β-glucan promotes the production of short-chain fatty acids (SCFAs), particularly propionate, by gut microbiota. An increased propionate-to-acetate ratio, resulting from fiber fermentation, reduces cholesterol biosynthesis, as acetate serves as a primary substrate for this process. Patents [89,90] explore formulations combining β-glucan with other cholesterol-lowering agents like plant sterols and/or soy protein, sometimes in combination with hydrocolloids or omega-3 fatty acids, to enhance the overall cholesterol-reducing effect and create functional foods or supplements. These patents highlight the potential for synergistic effects when β-glucan is combined with other bioactive compounds. While the cholesterol-lowering effects of oat β-glucan have been widely studied, further investigation is needed to clarify its impact on bile acid circulation. Additionally, the potential influence of oat β-glucan’s physicochemical characteristics—such as particle size, solubility, and conformation—on cholesterol metabolism represents a promising avenue for future research [91].

### 4.3. Modulation of Gut Microbiota

The gut microbiome supports host defense against various external stressors and contributes to overall health. Clinical trials have demonstrated that gut bacteria can mitigate several pathological conditions [92,93]. As a key prebiotic, β-glucan positively influences the gut microbiota, leading to beneficial physiological changes [94]. Researchers found that oat β-glucan forms a gel-like matrix in the gastrointestinal tract, increasing the viscosity of gastric and intestinal fluids [95]. This property, combined with its resistance to enzymatic digestion, inhibits pathogenic bacterial growth. Furthermore, oat β-glucan enhances the growth of beneficial bacteria like lactobacilli and bifidobacteria, while also supporting liver health. In the colon, it undergoes fermentation, particularly by cecal lactobacilli and bifidobacteria, promoting bifidobacterial growth and improving gut health. In a study with weaning piglets, Metzler-Zebeli et al. [96] found that oat β-glucan increased short-chain fatty acid (SCFA) production, leading to changes in gene expression. These findings underscore its prebiotic potential. Additionally, oat β-glucan has been shown to enhance insulin sensitivity and boost Na⁺K⁺-ATPase and Ca^2+^Mg^2+^-ATPase activity, particularly in the ileum [76].

### 4.4. Immunomodulatory Effects

β-Glucan is a natural immunomodulator that influences both innate and adaptive immune responses. It interacts with receptors such as C-type lectin receptors, complement receptor 3, lymphocyte surface antigens, and lactosylceramide, enhancing immune function [97,98]. Udayangani et al. [99] observed that nanoscale oat β-glucan (average size 465 nm) increased zebrafish larvae survival against pathogens and upregulated immune-related genes such as TNF-α, IL-1β, β-defensin, and lysozyme. The immunomodulatory effects of oat β-glucan are influenced by its molecular weight. Błaszczyk et al. [100] showed that high-molecular-weight oat β-glucan (2,180,000 g/mol) modulates inflammation-related gene expression more effectively in LPS-induced colitis in rats. In contrast, Wilczak et al. [101] found that low-molecular-weight oat β-glucan (70,000 g/mol) promoted short-chain fatty acid synthesis and enhanced antioxidant activity, demonstrating a stronger effect on these outcomes. Collectively, evidence from animal and cellular studies indicates that oat β-glucan regulates inflammatory cytokines, immune proteins, and inflammation-related pathways, thereby exerting its immunomodulatory effects.

### 4.5. Antitumor Activity

Cancer encompasses a wide range of diseases characterized by uncontrolled cellular proliferation, with malignant tumors capable of spreading to various body sites through the bloodstream or lymphatic system. Low-molecular-weight β-glucan derived from oats has demonstrated significant antitumor potential while remaining non-toxic to normal cells, making it a promising candidate for adjuvant or supplementary cancer therapy [102,103]. However, the high viscosity associated with high-molecular-weight β-glucan limits its therapeutic applications. Recent studies have shown that oat β-glucan exhibits potential anti-melanoma effects, which are further enhanced when combined with electroporation techniques [104]. It has also been used in breast cancer treatment and found to accelerate the resolution of radiation-induced inflammation in post-amputation sites [105]. Additionally, oral administration of β-glucan has been reported to augment the tumor-inhibitory efficacy of monoclonal antibodies in mice [106]. The anticancer effects of oat β-glucan appear to be mediated through the activation of adaptive immune cells, including CD^4+^ and CD^8+^ T cells and B cells. However, the detailed mechanisms of its anticancer activity remain complex, and the specific signaling pathways involved are not yet fully elucidated [107].

## 5. Modification and Functionalization of Oat β-Glucan

### 5.1. Acid Degradation

Common acids used for the acid degradation of oat β-glucan include hydrochloric acid, sulfuric acid, and phosphoric acid. Under acidic conditions, the glycosidic bonds break, resulting in a reduced molecular weight of oat β-glucan [108]. While acid degradation is straightforward and easy to perform, it has the drawback of producing a broad molecular weight distribution in the degraded products. Unlike oxidative degradation, acid degradation only shortens the molecular chains of oat β-glucan without affecting its hierarchical structure [109]. When oat β-glucan is degraded in a 0.2 M hydrochloric acid solution at 80 °C, the molecular weight of the product decreases with increasing degradation time [110]. Concurrently, the gel stability, elasticity, and strength of the β-glucan improve as the molecular weight decreases. Infrared spectroscopy shows no changes in absorption after degradation, and scanning electron microscopy reveals that as the molecular weight decreases, the microstructure of oat β-glucan transitions from dense and porous to loose, with the visible interlaced framework completely disappearing [111]. This indicates that only the glycosidic bonds in β-glucan are cleaved during degradation, without new functional groups being formed.

### 5.2. Oxidative Degradation

Oat β-glucan can undergo oxidative degradation in the presence of oxidizing agents such as hydrogen peroxide, sodium hypochlorite, and ascorbic acid. Among these, hydrogen peroxide is the most extensively studied because it degrades oat β-glucan non-toxically and without producing by-products [112]. Due to its strong oxidizing properties, hydrogen peroxide not only cleaves glycosidic bonds but also oxidizes exposed hydroxyl groups into carbonyl and carboxyl groups during the degradation process [113]. As the reaction time and hydrogen peroxide concentration increase, the content of carbonyl and carboxyl groups in oat β-glucan rises. This enhances the bile acid binding capacity of the degraded oat β-glucan and alters its swelling capacity. However, its gel viscosity, hardness, and adhesiveness decrease. This contrasts with the effect of acid degradation, which enhances the gel hardness of oat β-glucan [114]; the decrease in gel properties after oxidative degradation may be attributed to the formation of carbonyl and carboxyl groups. In addition to the use of oxidizing agents, lipid oxidation in food systems can also induce the degradation of oat β-glucan [115]. During lipid oxidation, β-glucan degrades, leading to reductions in its molecular weight and viscosity. Elevated temperatures accelerate this oxidation and further decrease viscosity. Therefore, the presence of β-glucan in food can delay lipid oxidation [115,116,117].

### 5.3. Acetylation Modification

Acetylated oat β-glucan is typically prepared by adding acetic anhydride to an alkaline β-glucan suspension, where acetyl groups replace hydroxyl groups to form acetylated β-glucan. According to FDA regulations, acetylated β-glucan with a degree of substitution between 0.01 and 0.20 is permitted for use in food products [118]. Research on how the degree of acetylation affects the functionality, structure, and rheological properties of oat β-glucan has shown that acetylation increases the heterogeneity of molecular degradation and promotes the formation of a dense, non-porous microstructure [119]. This modification enhances the swelling capacity and bile acid binding ability of β-glucan [120]. Compared to unmodified oat β-glucan gel, acetylated β-glucan gel exhibits reduced hardness and lacks adhesiveness, while its cohesiveness, elasticity, and viscosity are increased. Although acetylation facilitates gel formation, the viscosity of acetylated β-glucan decreases as the degree of acetylation increases [121]. Furthermore, acetylation can alter the biological activity of other types of β-glucans. For instance, site-selective modification of yeast β-glucan—achieving complete substitution of hydroxyl groups at the O-2 position and partial substitution at the O-4 position—has been found to enhance immune responses. The modified β-glucan, upon immune stimulation, interacts with mouse peritoneal macrophages, leading to increased phagocytic activity against *Escherichia coli* [122,123].

### 5.4. Sulfation Modification

Sulfation is one of the most commonly employed methods for modifying polysaccharides. The chlorosulfonic acid–pyridine technique is frequently used for this purpose. Due to the violent reaction of chlorosulfonic acid, it must be added dropwise into pyridine in an ice-water bath to prepare the esterification reagent [124]. Subsequently, the polysaccharide dissolved in dimethyl sulfoxide (DMSO) is placed in an ice-water bath, and the esterification reagent is slowly added [125]. The mixture is then heated to the desired temperature to carry out the esterification reaction. The reaction is terminated by adjusting the pH to 7.0, followed by alcohol precipitation, dialysis, and freeze-drying to obtain the sulfated polysaccharide [126]. Sulfated polysaccharides exhibit beneficial pharmacological properties such as antiviral and antitumor activities [127]. Their biological activity is influenced by several factors, including molecular weight, degree of sulfation, position of the sulfate groups, and water solubility. After sulfation, oat β-glucan shows decreased viscosity and increased water solubility. While its in vitro bile acid binding capacity decreases, its anticoagulant activity enhances with increasing concentration, possibly due to the high negative charge density of the sulfate groups [128].

Wang et al. [129] sulfated oat β-glucan using the chlorosulfonic acid–pyridine method to produce non-degraded, highly substituted sulfated β-glucan. This modified β-glucan demonstrated strong inhibitory effects against primary isolates of human immunodeficiency virus type I (HIV-I) in peripheral blood mononuclear cells (PBMCs) and also inhibited viral replication post-infection. Additionally, it protected PBMCs—pretreated by removing compounds—from infection, likely due to its high molecular weight and significant negative charge. These findings suggest that sulfated oat β-glucan could be a potential vaginal microbicide [130].

### 5.5. Carboxymethylation Modification

Carboxymethylation of natural polysaccharides like cellulose, starch, and glucans enhances their solubility, boosts biological activity, and broadens their application scope [131]. Although the carboxymethylation of oat β-glucan has not been reported yet, insights from other carboxymethylated β-glucans suggest potential improvements in its properties. For instance, carboxymethylation of fungal β-glucan induces macroscopic structural changes in the biopolymer, such as polymer disruption and surface bubble formation [132]. This modification also enhances the thermal stability and antioxidant capacity of fungal β-glucan and imparts antibacterial activity against *Candida tropicalis* [133]. Similar to sulfated polysaccharides, carboxymethylation alters the structure of polysaccharides, thereby enhancing the antioxidant and antibacterial activities of β-glucan.

Kagimura et al. [134] demonstrated that introducing carboxymethyl groups reduces intra- and intermolecular interactions, making fungal β-glucan more soluble and enhancing its antioxidant capacity. Additionally, the antioxidant activity of carboxymethylated derivatives increases with higher degrees of substitution [135]. Carboxymethylated yeast β-glucan has been shown to modulate vascular function and inhibit ADP-induced platelet aggregation, indicating its potential as an adjunct therapy for cardiovascular diseases [136]. Given that carboxymethylation enhances the functionality and applications of various β-glucans, further research into the property changes of carboxymethylated oat β-glucan is warranted.

## 6. Impact of Processing on Oat β-Glucan

After harvesting, oat grains undergo preliminary treatments such as dehulling and drying. They are then processed through methods like soaking, baking, steaming, and extrusion to produce various oat-based foods [137,138,139]. Alternatively, β-glucan can be extracted from oats and incorporated into foods as a functional ingredient. Heat processing, fermentation, extraction techniques, and the subsequent storage of oat products can all influence the molecular weight and viscosity of oat β-glucan to varying extents, resulting in changes to its physicochemical properties [140,141].

### 6.1. Effects of Thermal Processing

**Figure 5:**
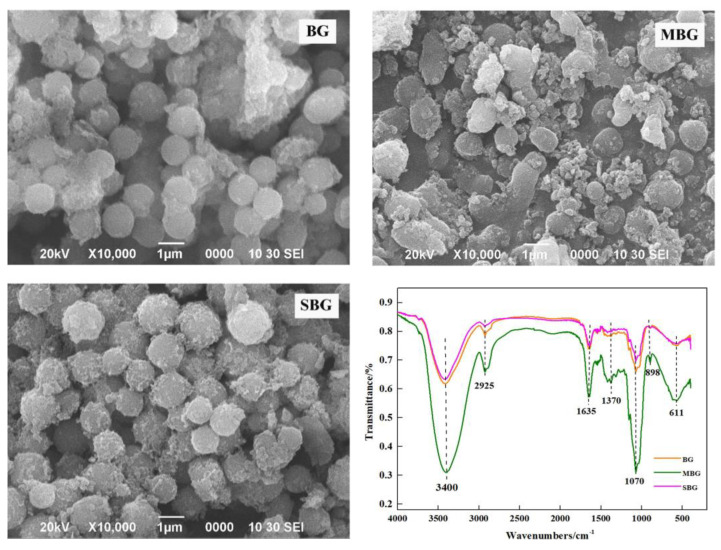
Scanning electron microscopy and FT-IR results for oat β-glucan processed using different thermal processes. BG, extracted β-glucan from whole grain oats without thermal processing; MBG, β-glucan from microwave-processed whole grain oats; SBG, extracted β-glucan from steam-processed whole grain oats [148].

Thermal processing influences the molecular weight and physicochemical characteristics of β-glucan (Figure 5). The viscosity of oat β-glucan decreases after dehulling and baking [142]. In contrast, drying oats increases the molecular weight and viscosity of β-glucan, possibly due to the inactivation of β-glucanase during drying [143]. Four common food processing methods—baking, boiling, steaming, and microwaving—affect the molecular weight of β-glucan in oat products to varying extents. Steaming causes the most significant degradation of oat β-glucan, leading to the greatest reduction in molecular weight, followed by boiling. Baking and microwaving result in the least degradation [144,145]. Compared to directly milling oat grains, steaming and milling or stir-frying and milling increase the extraction yield of β-glucan. Additionally, the viscosity of oat flour increases after steaming and milling or infrared roasting and milling, with the latter showing a more pronounced effect. This increase in viscosity may be attributed to the inactivation of β-glucanase by heat treatment [146,147].

### 6.2. Effects of Extrusion

**Figure 6:**
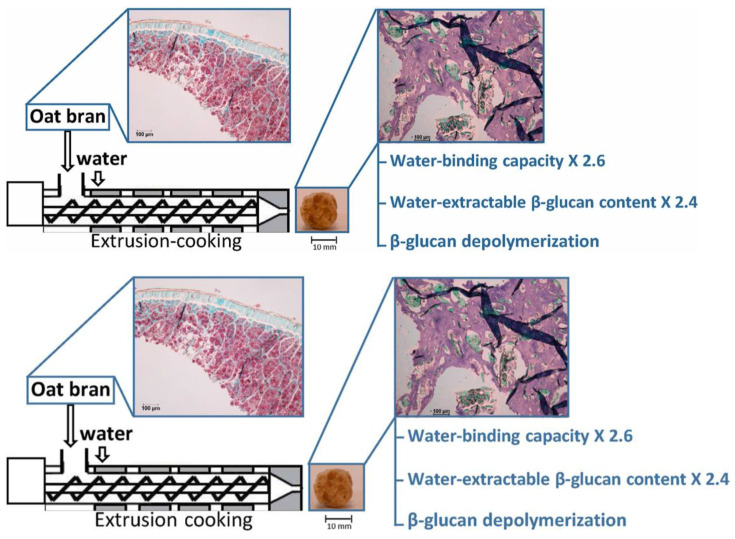
Extrusion increases oat β-glucan extractability [151].

Extrusion is another form of thermal processing that applies high temperature, pressure, and shear forces to food materials [149]. It can alter the molecular weight distribution of soluble dietary fiber (SDF) in oat bran and change the ratio of β-(1,3) to β-(1,4) linkages (see Figure 6). However, the effects of extrusion on the functionality, morphology, thermal properties, and rheological behavior of oat bran SDF are not yet fully understood. Compared to untreated oat bran, extruded oat bran SDF exhibits more aggregation, a higher gelatinization temperature, increased solubility and swelling capacity, and greater apparent viscosity, indicating that extrusion improves the functional properties of oat bran SDF [150]. Conversely, extrusion reduces the thermal stability of oat β-glucan [151]. This finding is supported by evidence that extrusion cooking, while increasing the water-extractable dry matter content and β-glucan extractability of oat bran, may not always preserve the original structure of the β-glucan molecule, potentially impacting its thermal stability and subsequent functionality. Furthermore, extruding gelatinized oat bran enhances the extraction rate and solubility of β-glucan, leading to a decreased degree of polymerization, which may affect its physiological activity [152]. Variations in extrusion parameters—such as temperature, moisture content, and screw speed—can differentially impact the properties of oat β-glucan [153].

### 6.3. Effects of Fermentation

The processing of foods often involves fermentation; however, certain fermentation processes can lead to the degradation of oat β-glucan, resulting in reduced molecular weight and viscosity [154,155,156]. In some cases, the larger particle size and shorter fermentation time of oat bran can limit β-glucan degradation, causing its molecular weight to remain relatively stable [145]. During the baking of oat bread fermented with yeast, β-glucan degrades, leading to a reduction in molecular weight, likely due to the presence of β-glucanase in the flour. Notably, increasing the degree of mixing and fermentation time can mitigate this degradation [145]. While some studies indicate minimal effects of fermentation on β-glucan content in oats, others highlight reductions under specific conditions or with particular microbial strains.

Recent research by Gupta et al. [157] indicated that the fermentation of oats using lactic acid bacteria did not significantly affect the β-glucan levels. In their study, fermented oat beverages showed average β-glucan contents ranging from 5.65% to 5.68%. Based on the findings of Bocchi et al. [158], the fermentation process did not significantly alter the oligosaccharide profile of oat milk during in vitro gastrointestinal digestion. This observation suggests that fermentation may preserve the prebiotic potential of oat β-glucan by maintaining its structural integrity and allowing for its subsequent utilization by beneficial gut microbiota in the colon. In contrast, Bernat et al. [159] noted a significant reduction of about 17% in β-glucan levels in oat milk fermented by specific bacterial strains. Additionally, studies suggest that the lack of added sugar during fermentation may lead to a marked decrease in β-glucan levels, possibly due to preferential consumption by Lactobacilli [160,161,162]. Further investigation is essential to understand the factors affecting β-glucan levels during fermentation and their implications for the nutritional value of fermented oats.

## 7. Application of Oat Beta-Glucan in Food Industry

Oat β-glucan exhibits multiple functional properties, including thickening, stabilization, emulsification, and gelling [163]. Recent research indicates that individuals with obesity, stemming from excessive fat consumption, face increased risks for conditions such as coronary heart disease, hypertension, stroke, diabetes, and cancer [164]. As a result, functional foods are viewed as a promising and safe approach for the management of these health issues, which has led to a significant interest in oat β-glucan within the food research sector. In various applications, including meat products, baked goods, sauces, soups, beverages, and others, oat β-glucan is predominantly employed for its emulsifying, thickening, stabilizing, and gelling characteristics to formulate functional foods [7].

### 7.1. Meat Products

**Figure 7:**
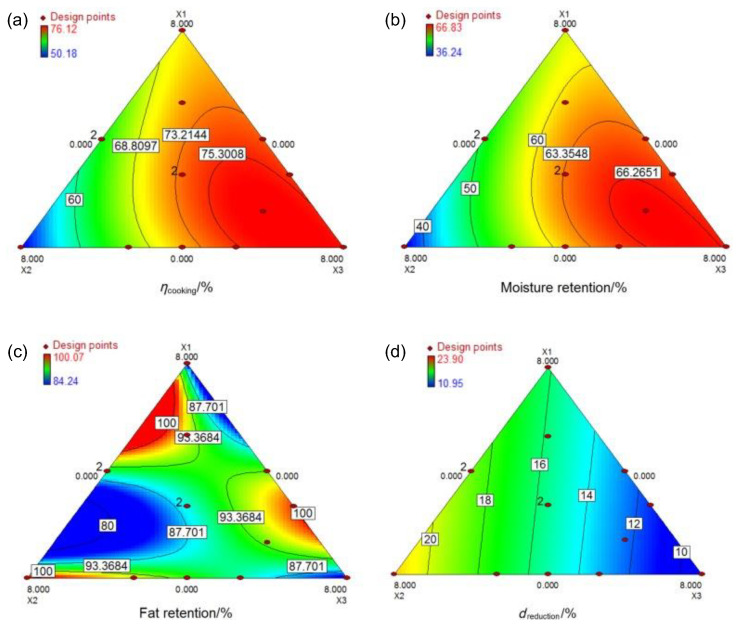
Contour plots for the effects of the addition of breadcrumbs (X1), inulin (X2), and β-glucan (X3) on (**a**) cooking yield, (**b**) moisture retention, (**c**) fat retention, and (**d**) reduction in the diameter of the low-fat beef burgers containing a canola and olive oil blend [170].

As public awareness of healthy diets continues to rise, the functional food market has evolved significantly since its inception in the mid-1980s, positively impacting the meat sector [93]. One approach to creating functional meat products involves incorporating ingredients such as prebiotics, which are known to enhance the activity of beneficial gut bacteria. Studies indicated that higher meat consumption is associated with an increased risk of colon cancer [165]; however, prebiotics and probiotics exhibit anticancer properties [166]. These beneficial compounds can reduce DNA damage in colon cells, lower the activity of pro-carcinogenic enzymes, inhibit the binding of mutagens, and boost immune responses [167,168]. Sarteshnizi et al. [169] found that oat β-glucan significantly improves the physical and sensory qualities of sausages, and its combination with resistant starch can lead to the production of probiotic sausages. Notably, their study demonstrated that the incorporation of resistant starch and β-glucan improved the moisture content of sausages while also exhibiting antioxidant properties, particularly in formulations containing lower levels of these prebiotic fibers. These findings highlight the potential of combining β-glucan with other prebiotic fibers to enhance the quality and stability of meat products while potentially providing additional health benefits. Additionally, Afshari et al. [170] demonstrated that β-glucan incorporation significantly enhanced the quality of hamburger patties, improving cooking yield, moisture retention, and sensory attributes such as acceptability and moldability (Figure 7). Notably, their study identified an optimal formulation that included a specific ratio of inulin to β-glucan, suggesting a potential synergistic interaction between these prebiotic fibers in enhancing the overall quality and functionality of meat products. Therefore, oat β-glucan serves as a valuable prebiotic ingredient in various meat products, improving not only product quality but also offering potential health benefits.

### 7.2. Beverage Products

Oat β-glucan is versatile, finding applications not only in cereal products but also in low-fat ice cream, yogurt, beverages, and various other food items [171,172]. Research indicates that incorporating oat β-glucan enhances the growth and activity of Lactobacillus in yogurt [173]. Rezaei et al. [174] demonstrated that β-glucan addition significantly enhanced the quality of frozen soy yogurt by improving its viscosity, texture, and stability while prolonging its shelf life. Their study showed that β-glucan increased viscosity, overrun, hardness, and fat destabilization while improving overall textural properties. Furthermore, the study highlighted the importance of aging time and temperature on product quality, with longer aging times at lower temperatures generally resulting in improved product characteristics. Ladjevardi et al. [175] reported that the inclusion of oat β-glucan increases probiotic viability, reduces fat content, and elevates the overall quality of yogurt. According to Mahrous et al. [176], oat β-glucan does not significantly alter the chemical composition of stirred and concentrated yogurt. Sharafbafi et al. [177] created low-calorie, low-cholesterol dairy products by adding high-molecular-weight oat β-glucan to milk. Rinaldi et al. [178] observed that yogurt containing both β-glucan and pectin exhibited faster protein breakdown and peptide release rates, along with a higher proportion of free amino acids compared to yogurt with starch and without β-glucan. Furthermore, Mielby et al. [179] found that oat β-glucan addition reduced the saltiness and clarity of tomato soup and the acidity of fruit beverages, though it did not significantly impact the aftertaste. Carmo et al. [180] proposed that oat β-glucan could effectively regulate the glycemic response to puffed snack products, highlighting its promising potential in the beverage industry. This proposition is supported by their findings, which demonstrated that extruded snacks containing oat β-glucan met the criteria for approved EFSA health claims related to cholesterol lowering and post-prandial glucose response. Notably, the study found that the extrusion process, while potentially inducing minor molecular weight changes in β-glucan, maintained sufficient β-glucan content to retain its functional properties, including the ability to modulate glycemic response.

### 7.3. Bakery Products

Incorporating oat β-glucan into bread and cake formulations enhances their physicochemical properties. Its inclusion in pasta is associated with a reduced glycemic index, thereby aiding in the management of metabolic diseases [181]. Baked goods containing oat β-glucan help slow glucose release, thereby reducing the risk of hyperglycemia. When β-glucan is added to wheat and millet composite breads, it mitigates quality defects resulting from the dilution and disruption of the gluten network, leading to improved volume, softer texture, and enhanced water retention, elasticity, and cohesiveness [182,183,184]. Furthermore, studies indicate that the addition of β-glucanase can lower the viscosity of β-glucan, which in turn enhances the gluten network structure and increases dough elasticity [185,186]. Ekstrom et al. [187] identified oat β-glucan as particularly suitable for baking due to its high molecular weight, which can also modulate the glycemic response to bread products. Oat β-glucan demonstrates superior rheological properties compared to barley β-glucan, resulting in higher-quality bread, potentially due to its greater viscosity [188]. Thus, the inclusion of oat β-glucan in baked goods not only improves their quality but also contributes to the development of functional baked products. EP 3045044 A1 describes a pregelatinized and ground flour containing β-glucan as a natural improver for food products, specifically for enhancing volume and structure and maintaining quality in baked goods [189]. This patent highlights the use of this improved flour in methods for producing dough and baked products with enhanced characteristics.

## 8. Limitations and Future Perspectives

Research on oat β-glucan has progressed significantly, revealing its multifaceted health benefits and diverse applications. However, several key areas warrant further exploration. First, elucidating the precise mechanisms underlying β-glucan’s diverse bioactivities remains a critical area of research. While studies have demonstrated its hypoglycemic, cholesterol-lowering, immunomodulatory, and prebiotic effects [190,191,192], the underlying molecular mechanisms remain incompletely understood. Further investigations are needed to elucidate how β-glucan interacts with gut microbiota, modulates immune responses, and influences host metabolic pathways [193]. This knowledge will not only deepen our understanding of β-glucan’s biological actions but also guide the development of novel β-glucan-based interventions for specific health conditions [194].

Furthermore, developing innovative and sustainable extraction [195,196] and modification [197,198] techniques is crucial for enhancing the cost-effectiveness and environmental sustainability of β-glucan production [199]. Emerging technologies such as supercritical fluid extraction [200], membrane filtration [201], and enzyme-assisted processes [202] offer promising avenues for improving extraction yields, reducing environmental impact, and producing β-glucan derivatives with tailored properties. Furthermore, research on green solvents [203] and bio-based catalysts [204] can contribute to the development of more environmentally friendly and sustainable extraction and modification processes.

Moreover, translating preclinical findings to human health outcomes requires further investigation. While animal studies and in vitro experiments have provided valuable insights into β-glucan’s potential health benefits [205,206], more robust human clinical trials are needed to confirm these findings and establish optimal dosage and consumption patterns for different populations [207,208,209]. Personalized nutrition approaches, considering individual gut microbiota composition [210], metabolic profiles [211,212], and genetic predispositions [213,214], may further enhance the efficacy and safety of β-glucan supplementation. Additionally, the development of novel β-glucan-enriched food products with improved sensory properties and consumer acceptance is crucial for their commercial success [215,216,217,218]. This necessitates a multidisciplinary approach involving food scientists, nutritionists, and sensory scientists to create innovative and appealing products that deliver the health benefits of β-glucan while meeting consumer demands.

Addressing regulatory and consumer concerns regarding the safety and efficacy of β-glucan-containing products will also be essential for their successful commercialization [7]. Clear and consistent regulatory guidelines, along with robust safety assessments and effective consumer education campaigns [219,220], will be crucial to build consumer trust and facilitate the widespread adoption of β-glucan-rich foods.

## 9. Conclusions

Oat β-glucan, a soluble fiber abundant in oats, has emerged as a promising functional food ingredient with diverse health benefits, including cholesterol-lowering, hypoglycemic, and immunomodulatory effects. This review comprehensively examines its extraction methods, structural characteristics, bioactivity, and applications in various food products. While significant progress has been made in understanding its properties and potential health benefits, several key challenges remain. Elucidating the precise mechanisms underlying β-glucan’s bioactivity, developing sustainable and cost-effective extraction and modification techniques, and translating preclinical findings to human health outcomes are crucial research priorities. Furthermore, addressing consumer acceptance, optimizing sensory properties, and navigating regulatory hurdles are essential for the successful commercialization of β-glucan-enriched foods. Interdisciplinary research involving food scientists, nutritionists, and other relevant disciplines is crucial to advancing the field of β-glucan research and translating its potential into impactful health solutions for consumers.

## Figures and Tables

**Table 1:** Hypoglycemic effects of oat β-glucan.

Type	Object	Period	Dosage	Effects	Ref
Human Experiment	Healthy subjects	24 h, washout 1 week	4 g oat β-glucan	Postprandial blood glucose and insulin ↓	[81]
Human Experiment	Diabetic patients	24 h, washout 6d	7.5 g oat β-glucan	Delayed gastric emptying, postprandial blood glucose and insulin ↓	[82]
Animal Experiment	Diabetic KM mice	6 weeks	800, 1200, 2000 mg/kg oat β-glucan	High-dose group showed the most significant effects on intestinal lactase, sucrase, and maltase activity ↓	[74]
Animal Experiment	Diabetic KM mice	6 weeks	2000 mg/kg oat β-glucan	Fasting blood glucose and glycosylated serum protein ↓; glycogen, hormones, and nuclear receptors ↑; free fatty acid content and succinate dehydrogenase activity ↓	[75]
Animal Experiment	SD rats	4 weeks	321.5 mg/kg oat β-glucan	Insulin sensitivity ↑; Jejunal Na^+^-K^+^-ATPase, Ca^2+^-Mg^2+^-ATPase activity ↑; Bifidobacterium and Lactobacillus ↑	[76]
Animal Experiment	Pigs	7 d	Oat β-glucan concentrate added to feed at 0%, 3%, 6%	Postprandial blood glucose, insulin, GIP, and GLP-1 ↓; SCFA levels ↑	[77]
Animal Experiment	Diabetic ICR mice	28 d	500, 1000, 2000 mg/kg OG500, 1000 mg/kg OG40, 1000 mg/kg OG7	Blood glucose ↓ and insulin resistance, and improving hepatic glycogen metabolism and islet tissue repair	[78]
Animal Experiment	Diabetic ICR mice	10 weeks	500 mg/kg oat β-glucan + 500 mg/kg L-arabinose, 1000 mg/kg oat β-glucan, 1000 mg/kg L-arabinose	Blood glucose and lipids ↓; insulin resistance ↑; GLUT4, PI3K, and P-AKT ↑; P-IRS1 protein ↓	[80]
Cell Experiment	IEC-6 cells	10–60 min	4, 6, 8 mg/mL oat β-glucan	SGLT1 and GLUT2 ↓; most significant in high-dose group	[79]

## Data Availability

No new data were created or analyzed in this study. Data sharing is not applicable to this article.
